# Mitochondrial Mutations are Associated with Atherosclerotic Lesions in the Human Aorta

**DOI:** 10.1155/2012/832464

**Published:** 2012-09-11

**Authors:** Igor A. Sobenin, Margarita A. Sazonova, Anton Y. Postnov, Yuri V. Bobryshev, Alexander N. Orekhov

**Affiliations:** ^1^Russian Cardiology Research and Production Complex, 121552 Moscow, Russia; ^2^Institute of General Pathology and Pathophysiology, Russian Academy of Medical Sciences, 125315 Moscow, Russia; ^3^Institute for Atherosclerosis Research, Skolkovo Innovative Centre, 143025 Moscow, Russia; ^4^School of Medical Sciences, Faculty of Medicine, University of New South Wales, Sydney, NSW 2052, Australia

## Abstract

Somatic mutations of the human mitochondrial genome can be a possible determinant of atherosclerosis. To test this possibility, forty mitochondrial mutations were analyzed in the present study in order to see which of these mutations might be associated with atherosclerosis. Ten mitochondrial mutations belonging to mitochondrial genes MT-RNR1 (rRNA 12S); MT-TL1 (tRNA-Leu, recognizes UUR); MT-TL2 (tRNA-Leu, recognizes CUN); MT-ND1, MT-ND2, MT-ND5, and MT-ND6 (subunits 1, 2, 5, and 6, respectively, of NADH dehydrogenase); and MT-CYB (cytochrome b) were potentially associated with atherosclerosis. From 29% (2 of 7 aortic samples) upto 86% (6 of 7 aortic samples) of aortic samples had a significant difference between atherosclerotic plaques and unaffected tissue, with the respect to the level of heteroplasmy for each mutation. Further, the homogenates of affected and normal intimae of 22 aortas were compared to reveal the average level of heteroplasmy for the above-mentioned 10 mutations. For five mutations, the mean level of heteroplasmy was significantly different in atherosclerotic intimal homogenates in comparison with the unaffected tissue. These mutations were A1555G, C3256T, T3336C, G13513A, and G15059A. Thus, it was demonstrated that at least five mitochondrial mutations occurring in MT-RNR1, MT-TL1, MT-ND2, MT-ND5, and MT-CYB genes are associated with atherosclerosis.

## 1. Introduction

Atherosclerosis underlies the development of most cardiovascular diseases, which are the leading cause of death in the 21st century. The mechanisms involved in the development of atherosclerosis have been intensively studied and various mechanisms and factors responsible for atherosclerotic alteration of the arterial intima have been suggested. Accumulating evidence supports an autoimmune mechanism as one of the prime pathogenic processes involved in the development of atherosclerosis [1–4].

 Recently we suggested that somatic mutations within the mitochondrial genome may be a probable cause of atherosclerosis development in humans [5]. In humans, the mitochondrial DNA (mtDNA) spans 16,569 DNA base pairs and is represented by a two-stranded circular molecule containing 37 genes. The two strands of mtDNA are differentiated by their nucleotide content, with the guanine-rich strand referred to as the heavy strand, and the cytosine-rich strand referred to as the light strand. The heavy strand encodes 28 genes, and the light strand encodes 9 genes. Of a total of 37 genes, 13 genes encode proteins (polypeptides), 22 genes encode transfer RNAs (tRNAs), and 2 genes encode the small and large subunits of ribosomal RNA (rRNA). Among the proteins, the subunits of complexes of a respiratory chain (cytochrome b, ATP synthase, cytochrome c oxidase, and NADH dehydrogenase) are encoded with mtDNA. Each mitochondrion contains several copies of its genome. Mitochondrial DNA is particularly susceptible to reactive oxygen species generated by the respiratory chain due to their close proximity. Though mtDNA is packaged by proteins and harbors significant DNA repair capacity, these protective functions are less robust than those functions operating on nuclear DNA and therefore are thought to contribute to the enhanced susceptibility of mtDNA to oxidative damage. In some cases, mtDNA mutations can cause maternally inherited diseases, and some evidence suggests that they may be major contributors to the aging process and age-associated pathologies. Mutations of mitochondrial DNA can lead to a number of illnesses, including exercise intolerance and Kearns-Sayre syndrome, which causes the loss of full function of the heart, eye, and muscle.

The penetrance and expression of mitochondrial mutations vary greatly between relatives and depend mainly on a genotype and the level of heteroplasmy (a mixture of mutant and normal molecules of DNA). Therefore, both a qualitative and a quantitative evaluation of a mutant allele of mitochondrial genome are necessary for studying the association of mitochondrial mutations with human diseases.

Studying associations between somatic mitochondrial mutations and focal development of atherosclerotic lesions in the intimal layer of human arteries is of high theoretical and practical impact. Such mutations may result in defects in the protein chains of respiratory enzymes and tRNAs that are synthesized in mitochondria, therefore producing oxidative stress and increasing the probability of plaque formation. However, the physical association of mitochondrial mutations with atherosclerotic lesions remains obscure. The present study was undertaken to test the hypothesis that several mitochondrial mutations can be associated with atherosclerotic lesions and, therefore, help explain the focal and mosaic nature of atherosclerosis development.

## 2. Methods

Thoracic aorta samples were collected 1.5 to 3 h after sudden death at the autopsy from 22 males and females aged between 23 and 70 years. The study was carried out in accordance with the principles outlined in the Helsinki Declaration of 1975, as revised in 1983. The protocol was approved by the ethics committee of the Russian Cardiology Research and Production Complex and by the ethics committee of the Institute of Atherosclerosis Research, Moscow.

The vessels were opened longitudinally and washed with phosphate-buffered saline (PBS), pH 7.6. The grossly normal parts of the arteries and those regions with atherosclerotic lesions were identified macroscopically and classified according to the classification of the Atherosclerosis Council of the American Heart Association [6, 7] utilizing the corresponding histological evaluations. Unaffected areas were defined as tissue samples with smooth luminal surfaces. Zones with initial atherosclerotic changes (type I lesions) corresponded to the parts of arteries with a smooth yellowish surface with occasional small yellow spots. Small aggregates of extracellular lipid droplets were present in the connective tissue matrix. According to the histology, apart from resident cells, the initial lesion foci were characterized by an increased number of mononuclear cells, in contrast to the visually intact intima. Fatty streaks (type II lesions) were defined as yellow strips and spots that slightly protruded over the vessel surface, often merging into larger structures and forming lesion clusters. In tissue sections, the presence of lipids was identified inside both macrophage-like cells and smooth muscle cells. The connective tissue matrix also contained extracellular lipids. Lipofibrous plaques (type Va lesions) were defined as spherical or elliptic protrusions of yellowish or nacreous color. Microscopically, they included accumulated intracellular lipids and increased amounts of extracellular matrix. Lipofibrous plaques contained a bulky necrotic core covered by a connective tissue layer and also included zones that morphologically resembling fatty streaks. Fibrous plaques (type Vc lesions) were defined as considerably protruding, rounded, or oval, and pearl-colored formations. They were mostly composed of a crude connective tissue matrix with embedded cells. The lipid component was rare. 

All analyzed 22 autopsy samples contained unaffected (nonatherosclerotic) zones which were estimated to constitute 10% to 45% of the luminal surface. All samples had zones with initial lesions and fatty streaks as well. Lipofibrous plaques were present in 12 aortic samples (55% cases) and occupied from 10% to 25% of luminal surface in these samples. Fibrous plaques were present only in 4 aortic samples (18% cases) and occupied from 3% to 12% of the luminal surface. Such a pattern of the distribution of atherosclerotic lesions throughout the luminal surface made it impossible, due to the low statistical power, to carry out an analysis of relation of heteroplasmy levels to the severity of atherosclerosis.

Homogenates of the affected (i.e., containing any above-mentioned lesion type or their combination) and normal intimae were compared to reveal an average level of heteroplasmy. To do this, all histologically verified segments of atherosclerotic intimae or unaffected regions were combined and homogenized, and after careful stirring, 10 *μ*g of tissue was taken for DNA extraction. 

DNA samples were obtained using commercially available kits for DNA extraction (BioRad, UK). For the amplification of fragments of mitochondrial DNA by polymerase chain reaction (PCR) method followed by pyrosequencing, the primers and conditions described elsewhere were used [7–25]. To quantitatively evaluate mutant alleles, a method of pyrosequencing [26–28] was adapted for conditions where both normal and mutant alleles were present in a biological specimen [25]. Briefly, the defective allele was quantified by analyzing the peak heights in the pyrogram of one-chained PCR fragments of a mitochondrial genome. The percent of heteroplasmy in DNA sample was calculated for each mutation, taking into account the expected sequence and the dimension of peaks for the homozygotes possessing either 100% of the normal or 100% of the mutant allele, as described elsewhere [25]. 

Statistical analysis was performed using SPSS v. 14 (SPSS Inc., USA). Wilcoxon statistics and frequency analysis were used for comparisons. The significance of differences was defined at a 0.05 confidence level.

## 3. Results

In this study we analyzed 40 mitochondrial mutations previously detected in such pathologies as coronary stenosis, some forms of diabetes, deafness, cardiac infarction, cardiomyopathy and stroke to reveal mutations associated with atherosclerosis [6–23]. At the first stage of this study we have analyzed DNA samples from segments of tissue from lipofibrous plaques and unaffected intimae of seven aortas. Thirty of analyzed mutations showed no difference in the level of heteroplasmy between atherosclerotic and normal tissues within the same aortic specimens. 

Ten mitochondrial mutations belonging to the following genes: MT-RNR1 (rRNA 12S); MT-TL1 (tRNA-Leu, which recognizes UUR); MT-TL2 (tRNA-Leu, which recognizes CUN); MT-ND1, MT-ND2, MT-ND5, and MT-ND6 (resp., subunits 1, 2, 5, and 6 of NADH dehydrogenase); mt-CYB (cytochrome b) were identified, which were unevenly distributed in aortic tissue, as from 43% (3 of 7) to 100% (7 of 7) aortic samples differed in the level of heteroplasmy for these mutations between atherosclerotic and normal tissues (data not shown).

Moreover, these mutations also appeared to be associated with atherosclerotic lesions because from 29% (2 of 7) upto 86% (6 of 7), aortic samples had a significant difference in the level of heteroplasmy for the given mutations in lipofibrous plaques in comparison with normal (unaffected) intimae. 

The demonstrated uneven distribution of mutations within aortic sample taken from single autopsy material could produce erroneous conclusion on the association of those mutations with atherosclerotic lesions due to random selection of tissue samples for mtDNA isolation. Therefore, further experiments compared PCR fragments of DNA extracted from the whole homogenates of the affected and normal intimae of all 22 aortas, focusing on the 10 mutations identified at the above stage of the study. Among these mutations, the level of heteroplasmy differed significantly in homogenates of affected and normal intimae for five of the mutations. These were single nucleotide substitutions A/G at position 1555, C/T at position 3256, T/C at position 3336, G/A at position 13513, and G/A at position 15059 (Table 1). The differences in the level of heteroplasmy did not reach statistical significance for nucleotide substitutions G/A at position 12315 and G/A at position 14459. Finally, there were no statistical difference in the level of heteroplasmy for mutations C/A at position 5178, G/A at position 14846, and InsG at position 652. The sample size (*n* = 22) was insufficient to provide valid examination of effects of confounding factors such as age, diabetes, and hypertension. However, regression and correlation analyses have been performed and showed that none of confounding factors possessed an explanatory value for heteroplasmy levels in the given data set.

Significant correlations were revealed between the levels of heteroplasmy for A1555G and C3256T (*r* = 0.365; *P* = 0.015), A1555G and T333C6 (*r* = 0.417; *P* = 0.005), A1555G and G15059A (*r* = 0.400; *P* = 0.007), between C3256T and T3336C (*r* = 0.407, *P* = 0.006), C3256T and G15059A (*r* = 0.667, *P* < 0.001), between T3336C and G13513A (*r* = −0.461, *P* = 0.002), between G5178A and G12315A (*r* = 0.380; *P* = 0.011), G5178A and G14459A (*r* = 0.325; *P* = 0.032), G5178A and G14846A (*r* = 0.800, *P* < 0.001), between G12315A and G14459A (*r* = 0.362, *P* = 0.016), G12315A and G14846A (*r* = 0.478, *P* = 0.001), and between G15059A and Ins652G (*r* = −0.487, *P* = 0.001).

## 4. Discussion

The association between mtDNA mutations and atherosclerotic lesions in the human aorta demonstrated by the present study is in agreement with a polygenic hypothesis of the origin and development of multifactorial diseases, which suggests that these pathologies may be the consequence of accumulated mutations. However, because some single mitochondrial mutations had higher prevalence in atherosclerotic tissue (i.e., the proportion of mtDNA copies bearing mutant allele was higher) and could possibly be the cause of the pathology, these results also support a monoclonal hypothesis of atherosclerosis. The last hypothesis considers the possibility of a somatic mutation appearing in a single smooth muscle cell that further proliferates and forms a monoclone; this monoclone could then expand into the vascular wall, followed by an intimal thickening and further development and growth of an atherosclerotic plaque [29]. It should be noted that the level of heteroplasmy for mutation G13513A was lower in atherosclerotic tissue as compared to unaffected aortic intima; this may allow offering a suggestion about atheroprotective role of this mutation which should be tested in further studies.

In contrast to comparisons of single lipofibrous plaques and unaffected intimal samples, in which the C5178A mutation seemed to be prevalent in normal tissue, the controversial results were obtained in comparisons of whole homogenates. There exists an assumption that C5178 mutation protects the intima from atherosclerosis [13]. However, our data do not confirm this assumption. In our research, the level of heteroplasmy for C5178A mutation has appeared to be lower in the whole homogenate of unaffected intima as compared to homogenates of atherosclerotic lesions.

On the basis of the obtained data, we conclude that at least five mitochondrial mutations, A1555G in MT-RNR1, G12315A in MT-TL2, G14459A in MT-ND6, C5178A in MT-ND2, and G15059A in MT-CYB are associated with atherosclerotic lesions in human aortic intima. Obviously, one of the limitations of our study is a lack of the demonstration of functional relationship between the presence of mtDNA mutations and the respiratory chain function (e.g., alteration of expression or enzymatic activities of respiratory complexes). However, it is worth to noting here that the investigation of functional relationship between the presence of mtDNA mutations and the respiratory chain function would require an independent expansive study.

Heteroplasmy is defined as the presence of a mixture of more than one type of an organellar genome within a cell or individual. Pathogenic mtDNA mutations are usually heteroplasmic, with a mixture of mutant and wild-type mtDNA within the same organism. A woman harboring one of these mutations transmits a variable amount of mutant mtDNA to each offspring. 

Heteroplasmy is common in humans and has been associated with aging and disease. Mitochondrial DNA is present in hundreds to thousands of copies per cell and also has a very high mutation rate. New mtDNA mutations arise in cells, coexist with wild-type mtDNA (heteroplasmy), and segregate randomly during cell division. The vast majority of deleterious mtDNA point mutations are heteroplasmic, and their mutant load can vary significantly among different tissues, even in the same subject. Heteroplasmic mtDNA defects are considered an important cause of human disease with clinical features that primarily involve nondividing (postmitotic) tissues. The amount of mutant mtDNA in a cell, called the heteroplasmy level, is an important factor in determining the amount of mitochondrial dysfunction and thus the disease severity. Both qualitative (presence or absence of a mutation) and quantitative (heteroplasmy level) estimations of mutant alleles in the mitochondrial genome are necessary for studying the association between mitochondrial mutations and human diseases, including atherosclerosis [5]. 

The cells that inhabit the subendothelial space in arteries participate in the processes of inflammation and atherosclerotic plaque formation. Increased levels of mtDNA heteroplasmy in arterial wall lead to a higher likelihood that cell function is inhibited due to the presence of mutations in the coding region of mtDNA. Impaired cell function, in turn, may lead to local oxidative stress and other pathologic events, which could promote atherosclerosis formation. Because free radicals and lipid peroxidation have been previously shown to be relevant in the etiology of atherosclerosis and coronary heart disease [30], among genetic factors, we hypothesize that mitochondrial mutations have a role in atherosclerosis [5]. 

## 5. Conclusion

Based on the data obtained in the present study, we now suggest that mtDNA heteroplasmy, which is a biomarker of defective mitochondrial function, can also be employed as a novel biomarker of atherosclerosis and consequent clinical manifestations of this disease.

## Figures and Tables

**Table 1:** Comparison of the level of heteroplasmy for ten mitochondrial mutations in homogenates of unaffected intimal samples and atherosclerotic lesions.

Mutation	The level of heteroplasmy (%)*		P**
	Unaffected tissue	Lipofibrous plaque	
652insG	3 (4)	1 (3)	NS
A1555G	13 (8)	20 (11)	0.001
C3256T	8 (4)	18 (7)	<0.001
T3336C	2 (2)	6 (5)	0.006
C5178A	12 (11)	15 (17)	NS
G12315A	15 (12)	20 (14)	0.069
G13513A	26 (7)	19 (11)	0.019
G14459A	5 (3)	7 (4)	0.054
G14846A	8 (6)	10 (9)	NS
G15059A	11 (11)	28 (14)	<0.001

*The level of heteroplasmy is expressed as a mean, SD is shown in

parentheses.

**The significance of differences was estimated by Wilcoxon signed-

rank test; NS: nonsignificant differences.
